# Activity Cliff-Informed Contrastive Learning for Molecular Property Prediction

**DOI:** 10.21203/rs.3.rs-2988283/v2

**Published:** 2024-12-04

**Authors:** Wan Xiang Shen, Chao Cui, Xiaorui Su, Zaixi Zhang, Alejandro Velez-Arce, Jianming Wang, Xiangcheng Shi, Yanbing Zhang, Jie Wu, Yu Zong Chen, Marinka Zitnik

**Affiliations:** 1 Department of Biomedical Informatics, Harvard Medical School, Boston, MA, USA; 2 Department of Chemistry, National University of Singapore, 117543, Singapore; 3 Tsinghua Shenzhen International Graduate School, Tsinghua University, Shenzhen, 518055, China; 4 Interdisciplinary Graduate Program in Integrative Biotechnology, Yonsei University, Incheon 21983, Korea; 5 Institute of Biomedical Health Technology and Engineering, Shenzhen Bay Laboratory, Shenzhen, 518055, China; 6 Kempner Institute for the Study of Natural and Artificial Intelligence, Harvard University, MA, USA; 7 Broad Institute of MIT and Harvard, Cambridge, MA, USA; 8 Harvard Data Science Initiative, Cambridge, MA, USA

**Keywords:** Activity Cliff, Contrastive Learning, Activity Cliff Awareness, Graph neural networks

## Abstract

Modeling molecular activity and quantitative structure-activity relationships of chemical compounds is critical in drug design. Graph neural networks, which utilize molecular structures as frames, have shown success in assessing the biological activity of chemical compounds, guiding the selection and optimization of candidates for further development. However, current models often overlook activity cliffs (ACs)—cases where structurally similar molecules exhibit different bioactivities—due to latent spaces primarily optimized for structural features. Here, we introduce AC-awareness (ACA), an inductive bias designed to enhance molecular representation learning for activity modeling. The ACA jointly optimizes metric learning in the latent space and task performance in the target space, making models more sensitive to ACs. We develop ACANet, an AC-informed contrastive learning approach that can be integrated with any graph neural network. Experiments on 39 benchmark datasets demonstrate that AC-informed representations of chemical compounds consistently outperform standard models in bioactivity prediction across both regression and classification tasks. AC-informed models show strong performance in predicting pharmacokinetic and safety-relevant molecular properties. ACA paves the way toward activity-informed molecular representations, providing a valuable tool for the early stages of lead compound identification, refinement, and virtual screening.

## Main

Molecular activity prediction is an essential part of drug discovery to identify, refine, and virtually screen compounds. It helps researchers evaluate the potential biological activity of chemical compounds and select lead candidates for development^1^. Ligand-based approaches use deep learning to build predictive quantitative structure-activity relationship (QSAR) models from datasets of molecular properties^1,2^. These models are designed to capture the complex relationships between molecular structures and their biological activities, utilizing a variety of molecular representations, such as graphs^3–5^, molecular descriptors^6–9^, images^10–13^, and fingerprints^14–16^. Geometric deep learning and graph neural networks (GNNs), which can directly model the structural and relational information of molecular graphs, have been successfully used for molecular activity prediction^17^. Despite their potential, GNNs can underperform compared to other methods^18–25^ and can perform worse than advanced molecular fingerprint techniques^19^. This drop in model performance can be in part attributed to the presence of activity cliffs (ACs)^19, 21, 26^.

ACs occur when structurally similar molecules exhibit drastically different biological activities, creating discontinuities in the chemical space that are difficult for models to capture accurately^27^. This issue is particularly problematic in GNNs, where the close embedding of structurally similar molecules in the latent space often leads to poor predictions when their activities differ significantly. Addressing the challenges posed by ACs is crucial for improving the accuracy and reliability of molecular activity predictions^19^. ACs not only complicate QSAR^27, 28^ modeling but also provide critical insights into the chemical and biological mechanisms underlying drug activity^29^. A distinct and more natural approach would be to handle ACs directly at the level of compound pairs, where models predict whether a matched molecular pair (MMP)^30^ forms an AC based on a predefined activity threshold (e.g., classifying as an MMP-cliff if the activity difference is greater than 100-fold, or as an MMP-nonCliff if the activity difference is less than 10-fold)^6, 13, 29, 31^. Studies indicate that QSAR regression models exhibit low AC sensitivity when the activities of both MMP compounds are unknown (i.e., absent from the training set), limiting their effectiveness for AC prediction^21^. Developing techniques to increase AC sensitivity could enhance the performance of QSAR models, providing a promising avenue for future research^21^.

Here, we introduce activity cliff awareness (AC-awareness), a novel inductive bias for GNN models designed to improve molecular activity prediction. AC-awareness enhances the model’s ability and sensitivity to distinguish structurally similar molecules with differing activities by optimizing the latent space to reflect these critical activity differences. Our approach optimizes the latent feature space to address discontinuities in structure-activity relationships (SAR) within the feature space^27^, avoiding fragmented feature distributions^32^. This is achieved by integrating a novel Activity Cliff Awareness (ACA) loss function, which combines a standard regression loss (e.g., MAE or MSE) with a triplet soft margin (TSM) loss. The TSM loss penalizes the model when the relative distances in the latent space between an anchor compound and its corresponding positive and negative compounds in high-value activity cliff triplets (HV-ACTs) do not reflect their activity relationships. To identify HV-ACTs, we introduce two parameters: cliff lower (cl) and cliff upper (cu). These parameters define the range of activity differences used to sample potential triplets during training, ensuring that the model focuses on the most informative triplets—those that challenge its ability to capture both activity differences and similarities.

GNN models trained using regression loss alone often fail to directly optimize high-value activity cliffs (HV-ACTs) in the latent space. In contrast, AC-informed GNN models that incorporate an AC-awareness bias generate a better-organized latent space. For instance, on the low-sample size and narrow scaffold (LSSNS) BRAF dataset, the model with AC-awareness improved label coherence in the latent space by 31.4% compared to the model without AC-awareness. This enhancement translated into average performance improvements of 7.54% and 21.6% over baseline models using MAE and MSE regression losses, respectively. Integrating AC-awareness into GNN models not only enhances performance across activity prediction tasks but also leads to more informative molecular representations, especially in datasets with prevalent activity cliffs. Experiments on nine LSSNS datasets and 30 higher-sample size and mixed scaffold (HSSMS) activity benchmark datasets demonstrate that incorporating AC-awareness can improve model performance by an average of 7.16% and 6.59%, respectively. Furthermore, AC-informed ACANET models outperform molecular fingerprint-based models on 70%−76% of the HSSMS benchmark datasets. The AC-awareness inductive bias is valuable beyond activity prediction tasks; we showcase its versatility by employing it to classify activity cliffs and predict other molecular properties, such as absorption, distribution, metabolism, excretion, and toxicity (ADMET). By enhancing the sensitivity of GNN models to property or activity cliffs, integrating AC-awareness demonstrates significant potential in property prediction tasks. Our results consistently show that models incorporating AC-awareness outperform state-of-the-art methods across various tasks and datasets, highlighting the promise of this approach in advancing molecular activity prediction.

## Results

### Equipping graph neural network models with AC-awareness.

**Figure 1:**
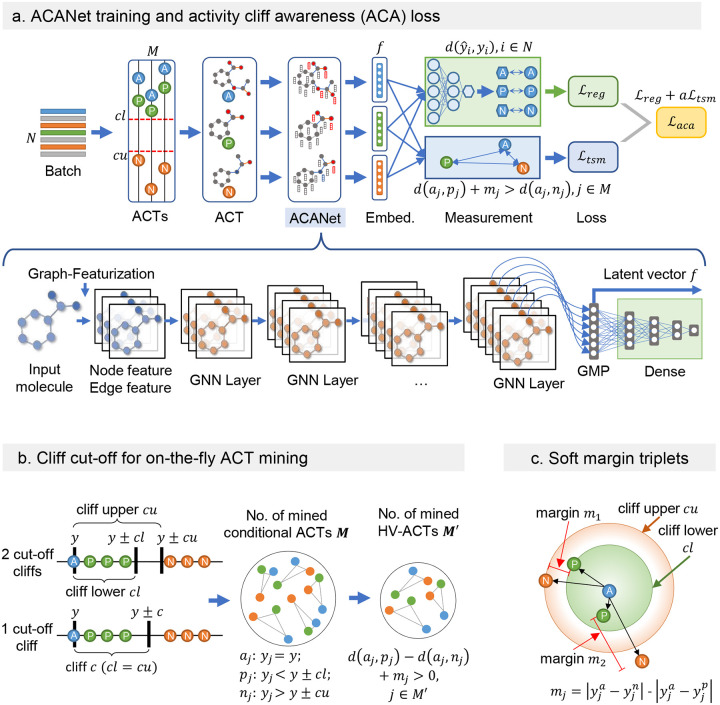
Overview of the proposed ACA loss and the GNN-based ACANET for molecular activity prediction. **(a) ACANET training and ACA loss**. During the training of ACANET, the ACA loss is calculated for each batch. The model dynamically mines activity cliff triplets (ACTs) in real time and distinguishes them in the latent feature space. **(b) Cliff cut-off scenarios for on-the-fly ACT mining**. Two cliff cut-offs, cliff lower (cl) and cliff upper (cu), are used to identify the positives (P) and negatives (N) for each given anchor (A) compound to obtain the conditional ACTs. One scenario is when the cl is equal to the cu. High-value (HV) ACTs are conditional ACTs with triplet loss greater than zero. **(c) Soft margin triplets in ACA loss**. Each triplet has a unique margin calculated from the ground truth labels rather than using a fixed margin for all triplets. These variable margins help the model adapt to the continuous activity labels in the regression task, improving its ability to accurately differentiate between compounds with similar and different activities.

The concept of AC-awareness was introduced to address the issues of ACs in GNNs used for molecular activity prediction. Here, the AC-awareness of a model is the ability to distinguish between structurally similar molecules with different activities in the learned latent space. The infusion of AC-awareness is achieved by minimizing the innovative ACA loss function, which balances regression loss (e.g., MAE) with triplet soft margin (TSM) loss, controlled by a tunable hyperparameter α. Figure 1a provides an overview of the proposed ACA loss and its implementation in the GNN-based ACANET model. The TSM loss penalizes the model when the relative distances between anchor (A), positive (P), and negative (N) compounds in the latent space deviate from their corresponding distances in the activity space. This penalty ensures that the model learns more informative features by aligning the latent space more accurately with the true activity relationships.

During the training of ACANET, the conditional ACTs are first mined using two cliff cut-off parameters—cliff lower (cl) and cliff upper (cu)—applied to the activity labels y, then only the highly value ACTs (conditional ACTs with their triplet loss values greater than zero) are used for the TSM loss calculation in each batch (Figure 1b). Each triplet in the TSM loss has a unique margin calculated from the ground truth labels rather than a fixed margin for all triplets (Figure 1c). These soft margins allow the model to handle continuous activity labels more effectively, improving its ability to differentiate between compounds with similar and different activities. Overall, the number of mined HV-ACTs reflects the degree of ACs in the current stage of learned molecular representations. If the model is equipped with AC-awareness during training, the number of mined HV-ACTs would be decreased gradually with the increase of training epochs. This is because the training objective of minimizing the TSM loss will reduce the number of mined HV-ACTs, helping the model focus on predicting latent feature space for more challenging compounds.

### Overview of datasets and tasks.

**Figure 2:**
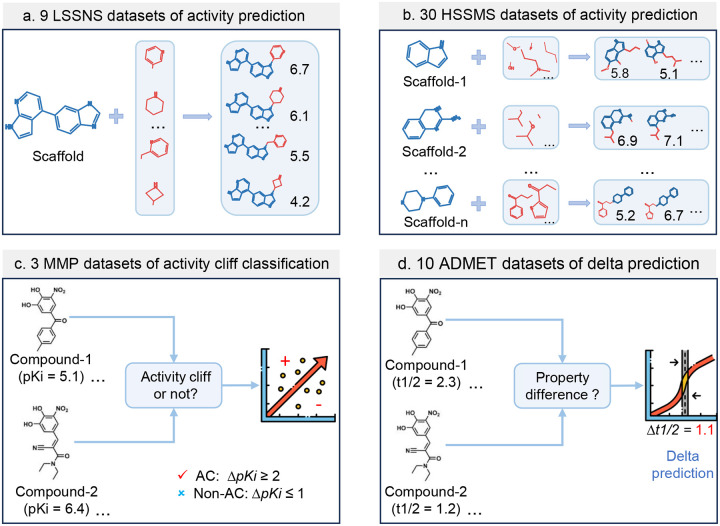
Schematic illustration of the datasets and tasks involved in this study. We evaluate the proposed method on four different cliff scenarios across 52 datasets. The first three scenarios involve protein target activity data, while the last pertains to ADMET data. The first two scenarios aim to predict the absolute activity values for a specific target, while the last two focus on predicting the relative differences of paired compounds. The third scenario involves the classification of cliff and non-cliff compounds, and the last scenario is a delta regression task. **(a) The nine low-sample size and narrow scaffold (LSSNS) datasets**. These datasets consist of compounds with limited and narrow scaffolds, typically developed by certain institutions or labs (Supplementary Table S1). These compounds are highly similar in structure, often being derivatives or analogs, but they exhibit significantly different activities to a specific target. **(b) The 30 high-sample size and mixed scaffold (HSSMS) datasets**. These 30 benchmark datasets were cleaned and compiled specifically to evaluate the activity prediction performance of ML models in cases with activity cliffs^19^. Originally integrated from multiple studies in the ChEMBL database, they comprise compounds with diverse scaffolds (Supplementary Table S2). **(c) The 3 MMP datasets of AC classification**. These three benchmark datasets (Supplementary Table S3)^31^ were generated by defining MMP as the MMP-cliff if $\Delta pK_{i}\geq 2$, and as MMP-nonCliff if $\Delta pK_{i}\leq 1$. **(d) The 10 ADMET datasets of delta prediction**. These benchmark datasets consist of three absorption datasets, two distribution datasets, an excretion dataset, three metabolism datasets, and a toxicity dataset (Supplementary Table S4). The tasks predict the ADMET delta values of paired compounds^38^.

We evaluated AC-awareness and the ACANET model across 52 datasets, grouped into four tasks (Figure 2b), to address the challenges posed by activity cliffs in molecular property prediction. These tasks represent distinct scenarios encountered in drug design. The first task focuses on predicting molecular activities within low-sample size and narrow scaffold (LSSNS, Supplementary Table S1) datasets, which include compounds with conserved scaffolds, often developed as derivatives or analogs. These nine datasets are representative of fragment-to-lead^33, 34^ and hit-to-lead^35^ drug design, where available data is limited. In this regime, researchers work with structurally similar compounds that share a core structure, aiming to optimize properties while maintaining or improving biological activity. However, ACs complicate QSAR analysis, making accurate molecular activity prediction more difficultThe second task involves predicting molecular activities using high-sample size and mixed scaffold (HSSMS, Supplementary Table S2) datasets. These 30 datasets contain diverse compounds with different scaffold types, allowing us to assess model performance in a broader chemical space^19^. These datasets represent midstage drug discovery, where models are developed to predict drug activity across multiple scaffolds through virtual screening^1^. Although HSSMS datasets provide more data and structural diversity, ACs still present significant challenges^19^, as they test the models’ ability to capture non-linear structure-activity relationships across a complex chemical space.

The third task is classifying activity cliffs using three Matched Molecular Pair (MMP) datasets. These datasets consist of pairs of structurally similar compounds with considerable differences in biological activity^21, 31, 36, 37^. This classification task is crucial in the lead optimization phase of drug discovery, as it helps develop structural alerts and avoid modifications that may result in substantial drops in activity. We extended AC-awareness to this classification task and tested its efficacy on these MMP datasets (Supplementary Table S3).

The fourth task involves predicting relative changes in 10 ADMET properties, referred to as delta prediction^38^. This task assesses how small modifications to a compound’s structure can influence its pharmacokinetic and safety profile. ADMET delta prediction is particularly relevant when minor changes in a molecule’s structure lead to considerable differences in ADMET profile. Accurately predicting these changes can help optimize a compound’s properties while minimizing the risk of adverse effects. The 10 ADMET datasets (Supplementary Table S4) were included to evaluate the broader applicability of our method beyond activity prediction.

### AC-informed models better capture activity cliffs than AC-agnostic models.

**Figure 3:**
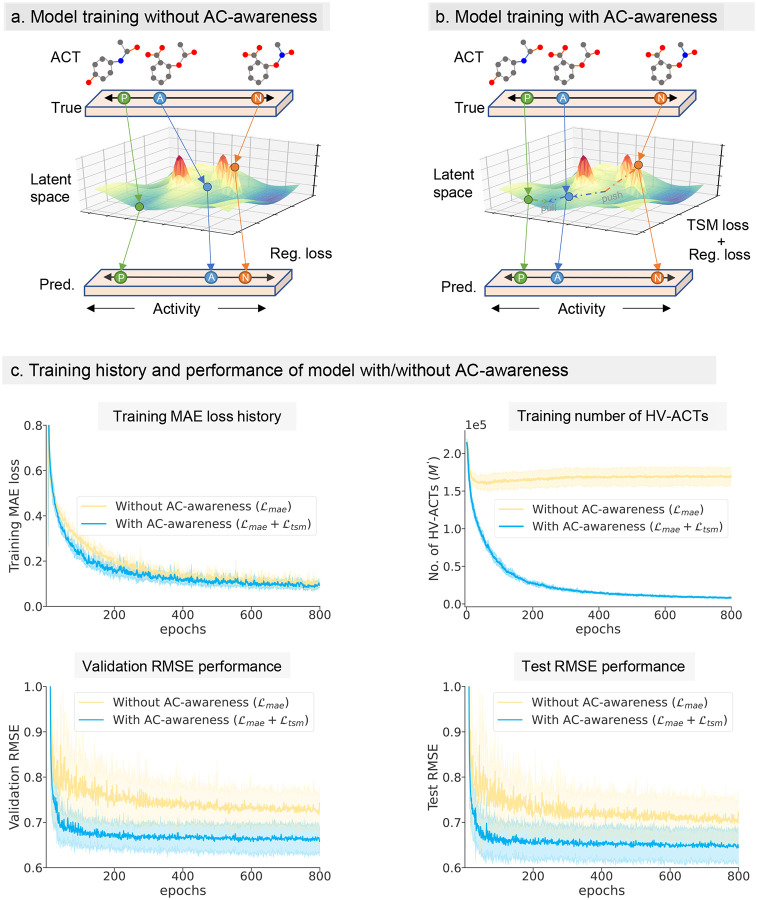
Comparison of model behavior and performance with or without AC-awareness on molecular activity prediction. **(a) Model training without AC-awareness**. Illustration of model training where the latent space does not account for activity cliffs (ACs). The learning process only optimizes for regression loss, leading to suboptimal differentiation between anchor (A), positive (P), and negative (N) samples. **(b) Model training with AC-awareness**. Illustration of model training equipping AC-awareness. The learning process includes triplet soft margin (TSM) loss $\left({ℒ}_{\text{tsm}}\right)$, which helps better differentiate between A, P, and N samples in the latent space in addition to regression loss $\left({ℒ}_{\text{mae}}\right)$. **(c) Training history and performance of PNA-based ACANET model with/without AC-awareness**. Comparison of models with AC-awareness (α=1) and without AC-awareness (α=0, no TSM loss) over training epochs. The plots show training MAE loss, number of mined HV-ACTs $\left(M^{\prime }\right)$, validation RMSE, and test RMSE of PPARδ dataset (n = 1125) of HSSMS. Shaded areas represent the standard deviation from 10 repetitions with different random seeds in dataset split.

Figure 3a and b presents a comparison between a GNN model trained without (left) and with (right) AC-awareness. To illustrate the challenge posed by activity cliffs (ACs), consider an activity cliff triplet (ACT) consisting of an anchor compound (A), a positive compound (P), and a negative compound (N). Although A and P are much closer in activity than A and N, A and N are more structurally similar, exemplifying the AC issue. Without AC-awareness, the model learns representations where the distance between A and P is much greater than between A and N in the latent space. This occurs because A and N exhibit high structural similarity despite their differing activities. Consequently, the model struggles with accurate training and prediction outcomes, facing challenges in effectively learning within the latent space due to AC issues. Conversely, with AC-awareness, the model addresses this challenge by optimizing the latent vectors, ensuring that A is closer to P and further from N. In other words, the ACA loss function encourages the model to learn a latent space where the relative distances between compounds correspond to their activity relationships. Overall, the model with AC-awareness integrates metric learning in the latent space while minimizing regression error. In contrast, a model without AC-awareness focuses solely on regression loss and fails to perform well in the presence of ACs.

To validate the effectiveness of AC-awareness, we conducted a study using a medium-size PPARδ dataset from the diverse HSSMS collection. Initially, we used the fixed ACA loss parameters (α=1,cu=1,cl=1) to test the differences in training behavior, validation, and test performance between models with AC-awareness (MAE loss + TSM loss) and without AC-awareness (MAE loss only) (Figure 3c). The results show that models with AC-awareness tend to converge faster (training MAE loss), although the final MAE loss is not significantly different between the two models. However, the optimization of HV-ACTs during training varied greatly. In the model without AC-awareness, the number of HV-ACTs remained almost constant throughout training. In contrast, the model with AC-awareness showed a gradual decrease in HV-ACTs during training, indicating that the model was refining the latent space to overcome the challenges posed by ACs. Remarkably, the validation and test RMSE results further highlighted the superiority of AC-awareness. When generalized to external validation and test sets, models with AC-awareness consistently achieved significantly lower RMSE values than those without AC-awareness, demonstrating the substantial benefits of incorporating AC-awareness into the training process.

**Figure 4:**
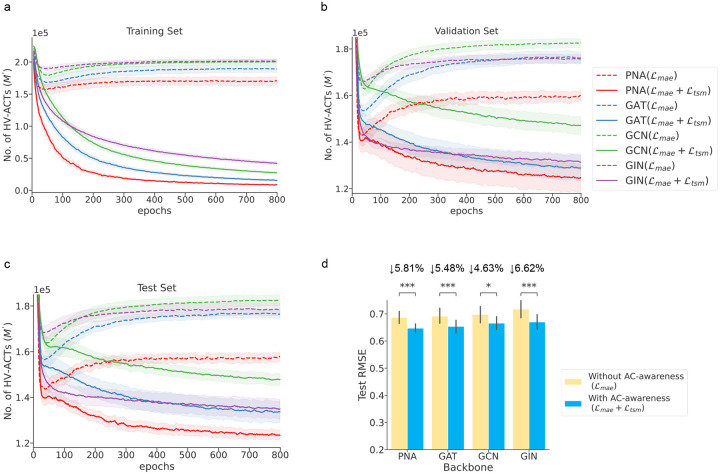
Impact of AC-awareness across different GNN backbones. The plots show the number of mined HV-ACTs $\left(M^{\prime }\right)$ during model training and the final test RMSE on the PPARδ dataset. The medium-sized PPARδ dataset (n = 1125) from the HSSMS collection was split into training, validation, and test sets in a 6:2:2 ratio, repeated ten times with different random seeds. The ACANET models (with four different backbones) were trained with and without AC-awareness, using identical hyperparameters except for the loss function: ${ℒ}_{\text{mae}}$ for models without AC-awareness and ${ℒ}_{\text{mae}}+{ℒ}_{\text{tsm}}$ for models with AC-awareness. **(a, b, and c) Number of mined high-value activity cliff triplets (HV-ACTs) in the training, validation, and test sets across four GNN backbones, respectively:** Graph Convolutional Network (GCN), Graph Isomorphism Network (GIN), Graph Attention Network (GAT), and Principal Neighborhood Aggregation (PNA). Dashed lines represent the training process without AC-awareness, while solid lines represent the training process with AC-awareness. The significant decrease in the number of HV-ACTs in models with AC-awareness indicates improved learning and handling of activity cliffs as the latent feature space is optimized. **(d) Final test set RMSE performance across the same GNN backbones, comparing models trained with and without AC-awareness**. The test RMSE performance is based on the model selected by the validation set RMSE. The numerical RMSE values for the test and validation sets with and without AC-awareness are provided in Supplementary Table S6. The equipping of AC-awareness consistently reduces RMSE across all backbones, with statistical significance indicated by * (*=p<0.05, **=p<0.01, ***=p<0.001, ****=p<0.0001).

The ACANet model uses PNA as its default backbone, but we also tested it with three other GNN backbones: GCN, GAT, and GIN. The results, shown in Figure 4, indicate that across all tested GNN backbones, models equipped with AC-awareness exhibit a gradual decrease in HV-ACTs during training, which also generalizes to the validation and test sets. Performance improvements were observed across the board, with test RMSE reductions of 5.81%, 5.48%, 4.63%, and 6.62% for the PNA, GAT, GCN, and GIN models, respectively, compared to models without AC-awareness. These findings strongly support the effectiveness of AC-awareness in enhancing the predictive performance of the model by effectively handling the challenge of activity cliffs, regardless of the GNN model backbone used.

### Impact of ACA loss hyperparameters on molecular activity prediction.

While the above analyses confirm the effectiveness of AC-awareness, the influence of hyperparameters in the ACA loss requires further investigation. We first conducted two ablation experiments to evaluate the impact of the ACA factor α and cliff parameters (cu and cl) in the ACA loss (with MAE as regression loss) on ACANET model (PNA backbone) performance. We also explore the improvements of AC awareness on activity prediction performance under different regression losses.

**Figure 5:**
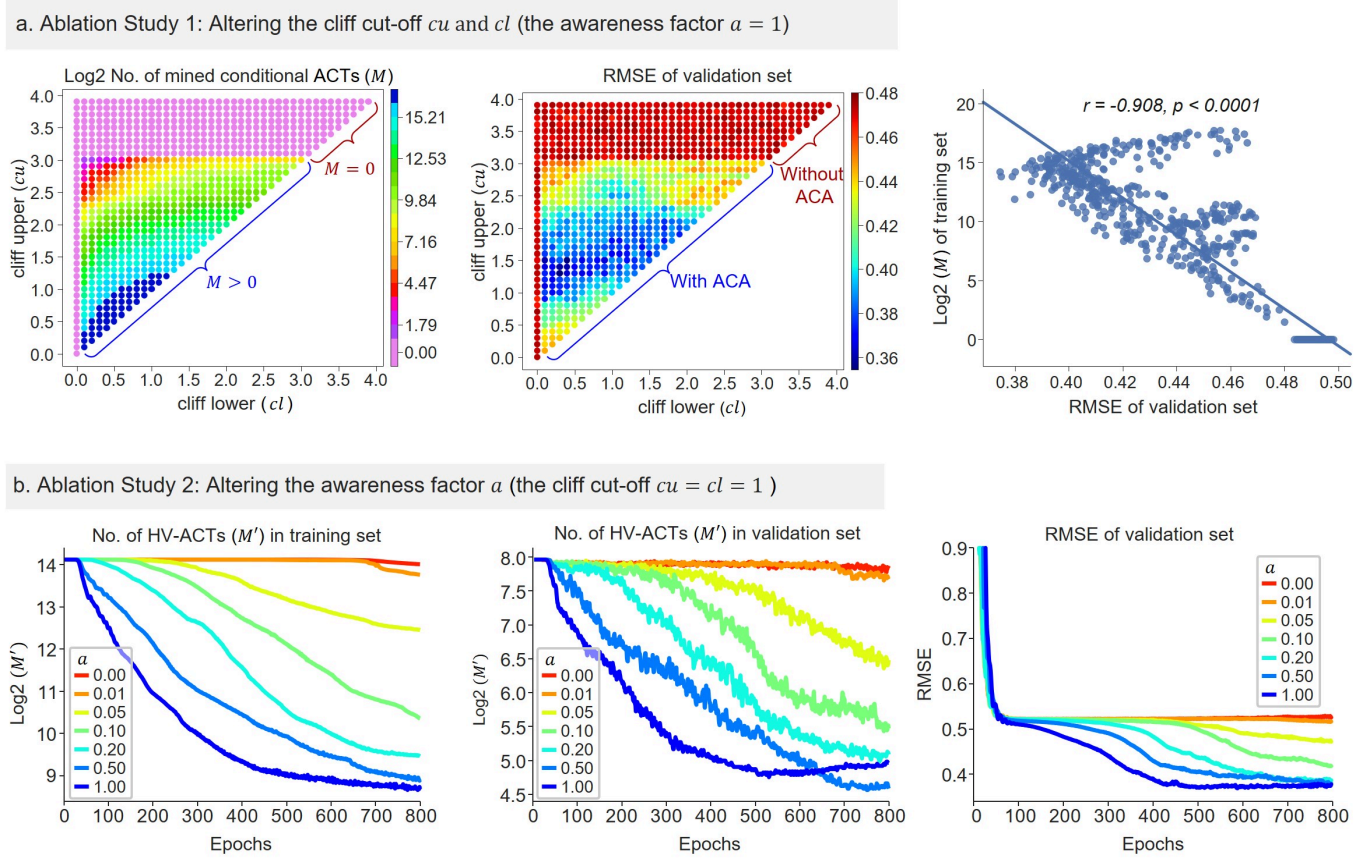
Ablation studies on the impact of cliff cut-offs and the awareness factor on model performance in the BRAF dataset. **(a) Shown are the effects of varying the cliff cut-off parameters** cl **and** cu **while keeping the awareness factor** α **fixed**. The number of mined conditional ACTs (M) and validation RMSE are displayed, illustrating how different cl and cu values affect the number of conditional ACTs M and model performance. The pink dots indicate where the number of conditional ACTs M is zero, essentially representing cases without AC-awareness. There is a clear negative correlation between the number of conditional ACTs in the training set and validation RMSE performance. The correlations between the number of conditional triplets M in the training set and validation RMSE performance for the other four LSSNS datasets (PLK1, IDO1, USP7, and RIP2) are shown in Supplementary Figure S2. **(b) Examines the impact of varying the awareness factor** α **while keeping** cl **and** cu **constant**. The results show the number of HV-ACTs and validation RMSE over training epochs for different α values, highlighting improved performance with higher α values.

In the first ablation study (Figure 5a), we varied the cliff parameters while keeping awareness factor α as 1.0 to assess their impact on the number of mined conditional ACTs (M) and the RMSE performance of ACANET. The number of mined ACTs (M) is determined by the label distribution and the cliff parameters, with a maximum M achievable under specific cl and cu settings, which can vary by dataset (Supplementary Figure S1). When M=0 (represented by the pink dots in Figure 5a), the TSM loss is zero, and the ACA loss is determined solely by the MAE loss, leading to a significant decrease in model performance. Conversely, when M>0 (i.e., with AC-awareness), the model performance improved significantly. Although both the quality and quantity of mined ACTs contribute to model performance improvement, the RMSE showed a highly negative correlation with the number of mined ACTs (Supplementary Figure S2), indicating that the quantity of ACTs significantly affects model performance. This study highlights the importance of selecting appropriate cliff cut-off parameters to efficiently mine ACTs and enhance cliff awareness and model performance.

In the second ablation study (Figure 5b), we examined the impact of the AC-awareness factor α on the effectiveness of the model performance in molecular activity prediction. We fixed the cliff parameters (cl and cu) and varied α from 0.00 (no cliff awareness) to 1.00. The results showed that increasing α led to a decrease in the number of mined HV-ACTs $\left(M^{\prime }\right)$ in both the training and validation sets, indicating improved cliff awareness (Figure 5b). Conversely, when α=0, the number of mined HV-ACTs remained almost unchanged during training, indicating no cliff awareness, consistent with our findings. Validation RMSE decreased as α increased, with greater improvement in performance observed for larger values of α. However, α represents a trade-off between the normal regression loss and the triplet contrastive loss in the ACA loss function. Setting α too high may overemphasize the triplet contrastive loss, resulting in overfitting and decreased performance. Therefore, a careful hyperparameter search is necessary to determine the optimal value of α for a given task and dataset. Our results suggest that selecting appropriate cliff parameters and α can enable efficient cliff awareness in molecular activity prediction, leading to better model performance.

We evaluated the impact of AC-awareness in training ACANET models with different regression loss functions, specifically focusing on PNA-based architectures. The cross-validation performance on the LSSNS datasets and the optimized parameters (α,cl,cu) are provided in Supplementary Table S7. Our findings indicate that incorporating AC-awareness significantly enhances model performance, regardless of whether MAE or MSE is used as the regression loss function. In baseline activity prediction tasks on the LSSNS datasets, MAE outperforms MSE. However, after integrating AC-awareness, the improvements with MSE are particularly noteworthy. Across the nine LSSNS datasets, models using MAE as the baseline with AC-awareness demonstrated an average improvement of 7.16%, while those using MSE showed a more substantial average improvement of 17.23%. The pronounced improvement observed with MSE can be attributed to its sensitivity to large errors, as MSE disproportionately penalizes large errors associated with activity cliffs. By incorporating AC-awareness, the model becomes better at detecting and addressing these cliffs, leading to a reduction in the large errors that MSE would typically amplify. This results in greater performance gains when using MSE compared to MAE. Nevertheless, despite the considerable benefits MSE derives from AC-awareness in mitigating large errors, models using MAE in combination with AC-awareness ultimately achieve superior overall performance (Supplementary Table S7). The linear treatment of errors by MAE, combined with the robustness introduced by AC-awareness, provides a more balanced and effective approach for molecular activity prediction across the datasets.

### Using AC-awareness enhances activity-coherent latent molecular representations.

**Figure 6:**
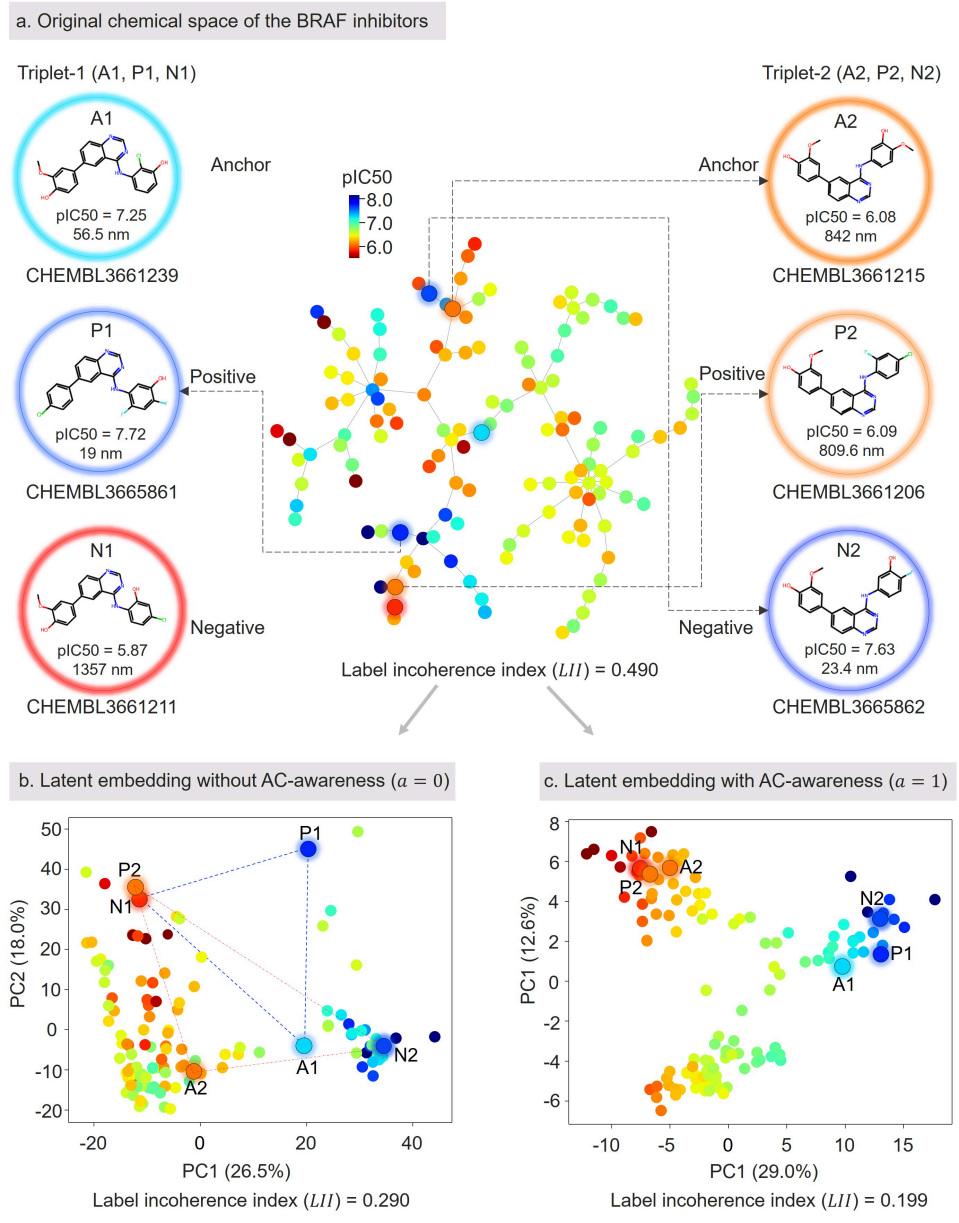
Comparison of original chemical and latent space learned by models with/without AC-awareness in the BRAF dataset. **(a) Original chemical space of the BRAF dataset**. Two example triplets (Triplet-1 and Triplet-2) are shown with their anchor, positive, and negative compounds. The compounds are color-coded based on their pIC50 values. Label incoherence index (LII)=0.490. **(b) Latent embedding without AC-awareness** (α=0). Principal Component Analysis (PCA) of the latent space without AC-awareness, showing poor separation between anchor/positive and negative samples. Label incoherence index (LII)=0.290. **(c) Latent embedding with AC-awareness** (α=1). PCA of the latent space with AC-awareness, showing better separation between anchor/positive and negative samples. Label incoherence index (LII)=0.199.

In molecular representation learning, optimizing the latent space can sometimes fail to capture shared molecular features, leading to fragmented feature distributions^32^. This fragmentation becomes particularly problematic in regression tasks involving ACs, where structurally similar molecules may be mapped to nearby regions in the latent space despite having significantly different activity labels. This misalignment leads to incoherent label distributions and diminished model performance. The issue is especially evident in graph-based molecular representations, where topological information, while useful for capturing structural similarities, can complicate the distinction between ACs. For example, in the first activity-cliff triplet (Triplet-1) shown in Figure 6, a high-potency anchor (A1, CHEMBL3661239) and a positive compound (P1, CHEMBL3665861) with IC50 values of 56.5 nM and 19 nM against BRAF, respectively, are expected to be closely positioned in the latent space (Figure 6a). However, in the GNN model without AC-awareness, the distance between A1 and P1 was not shorter than the distance between A1 and the negative compound (N1, CHEMBL3661211), which has a similar structure but significantly reduced bioactivity (IC50 of 1357 nM). Similarly, in the second activity-cliff triplet (Triplet-2), the distance between a lower-potency anchor (A2, CHEMBL3661215, IC50 of 842 nM) and a positive compound (P2, CHEMBL3661206, IC50 of 809.6 nM) was not closer than that between A2 and the negative compound (N2, CHEMBL3665862, IC50 of 23.4 nM), which exhibits higher bioactivity despite its structural similarity to A2. These examples highlight the model’s inability to properly handle ACs without AC-awareness, leading to suboptimal latent space distributions (Figure 6b). In contrast, with AC-awareness, the model successfully pushes N1 and N2 further away from their respective anchors (A1 and A2) and positive compounds (P1 and P2), refining the latent space to better handle ACs (Figure 6c). These observations underscore the importance of AC-awareness in guiding the model to prioritize bioactivity differences over structural similarities when mapping compounds in the latent space. By incorporating AC-awareness, the model becomes more effective at distinguishing between compounds with similar structures but differing bioactivities, optimizing the latent space distribution and improving the accuracy of activity predictions.

We computed the label incoherence index (LII) for the BRAF dataset before and after model learning to analyze the coherence of activity label distributions in the original chemical space and the learned latent space. We also visualized the label distributions in the original chemical space and the latent space learned by models with and without AC-awareness (Figure 6). The label incoherence index measures the degree of activity label disparity among neighboring molecules; a lower LII indicates higher consistency between molecular distances and their activity labels. Our findings show that, compared to the original chemical space, the LII decreased in the latent spaces learned by the models. Notably, the latent space learned with AC-awareness exhibited a significantly lower LII (0.199) than that without AC-awareness (0.290), indicating higher coherence. This improvement is also evident in the qualitative analysis of label color distribution, where the space with AC-awareness shows greater label continuity (Figure 6). In conclusion, our results demonstrate that the incorporation of AC-awareness significantly improves label coherence in the latent space, highlighting its crucial role in enhancing the model’s ability to distinguish bioactivity differences effectively.

### AC-informed ACANET outperforms other methods on HSSMS.

We evaluated the performance of ACANET with ACA loss on the 30 HSSMS benchmark datasets for molecular activity prediction. In the benchmark study by Van Tilborg *et al*. (2022)^19^, various models were assessed, including graph-based GAT, GCN, AFP, and MPNN; SMILES string-based Transformer, CNN, and LSTM; and fingerprint ECFP-based MLP, KNN, GBM, RF, and SVM. Among these, ECFP-based conventional ML models, particularly the SVM, achieved state-of-the-art (SOTA) performance. We compared the performance of these algorithms and ACANET on the overall test set $RMSE_{\text{all}}$ and the cliff-specific test set $RMSE_{\text{cliff}}$ of the HSSMS datasets using RMSE as the evaluation metric. The cliff parameters and AC-awareness factor α were optimized using a linear search under nested five-fold cross-validation (illustrated in Supplementary Figures S3), as described in the Methods section.

**Figure 7:**
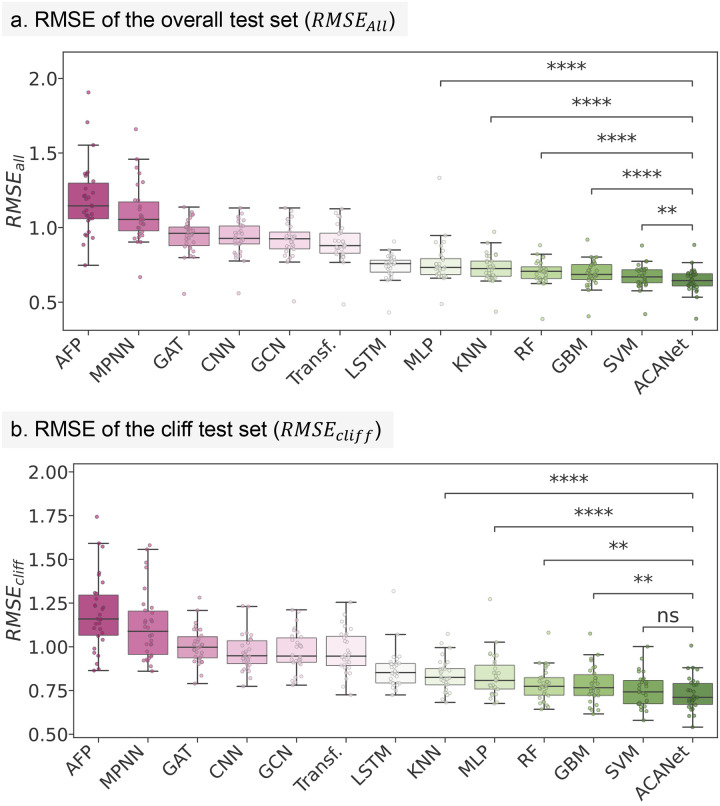
Performance comparison of ACA loss-based ACANET against shell-on-head ML approaches on the 30 HSSMS benchmark datasets. **(a)** Boxplot of RMSE performance on the entire test set of the 30 HSSMS datasets, where lower RMSE values indicate better performance. **(b)** Boxplot of RMSE performance on the cliff-specific test set of the 30 HSSMS datasets, where lower RMSE values indicate better performance. The significance of the RMSE improvements by ACANET compared to conventional ML models (SVM, RF, GBM, MLP, and KNN) using ECFP as input was evaluated using paired t-tests. Statistical significance is indicated by asterisks (*=p<0.05,**=p<0.01,***=p<0.001,****=p<0.0001,) or “n.s.” if not significant.

These results showed that ACANET consistently scored much lower RMSE values on both the overall test set and the cliff-specific test set (Figure 7). The superior performance of ACANET can be attributed to the equipment of AC-awareness, as evidenced by the impact of the factor α on the model’s performance across various datasets (Supplementary Figure S5). The model with AC-awareness successfully reduced the cross-validation RMSE across all 30 HSSMS datasets, achieving an average performance improvement of 6.59% across all datasets (Supplementary Table S8). On $RMSE_{\text{all}}$, ACANET outperforms all of the fingerprint ECFP-based ML approaches significantly. In particular, ACANET outperformed the SOTA ECFP-based SVM (Supplementary Figure S6) by notable margins in both $RMSE_{\text{all}}$ (0.646 vs 0.671) and $RMSE_{\text{cliff}}$ (0.711 vs 0.742) across all 30 datasets. In terms of individual datasets, ACANET outperformed the SOTA SVM for 23 and 21 of the 30 datasets in $RMSE_{\text{all}}$ and $RMSE_{\text{cliff}}$, respectively. These results suggest that ACANET with ACA loss is a robust approach for predicting the activity of both overall and cliff-specific compounds in the HSSMS datasets. We further analyzed the impact of the AC-awareness factor α on the performance of a more challenging dataset, CLK4, and an easier dataset, 5-HT1A^19^. We observed that a larger α value contributed to better performance on CLK4, whereas for 5-HT1A, a larger α tended to result in overfitting (Supplementary Figure S7). This occurs because a larger α emphasizes the ACA loss, penalizing the model for not correctly predicting the ACs. In an easier dataset like 5-HT1A, ACs may not play as crucial a role in overall bioactivity prediction. Thus, a larger α may force the model to overly focus on ACs at the expense of overall performance. Therefore, the tunable α parameter helps the model adapt to different situations and ensures robustness across various datasets.

### AC-awareness can help classify activity cliffs.

We extended the concept of AC-awareness to classification tasks for predicting AC and non-AC pairs (Supplementary Table S3). Unlike absolute regression activity prediction in QSAR models, MMP-based AC classification aims to determine whether a pair of similar compounds form an AC^21, 31, 36, 37^. In contrast to regression tasks, AC-awareness in classification is derived from the contrastive learning of paired molecules in latent space by pushing the molecules of AC pairs further apart and bringing the molecules of non-AC pairs closer together (as detailed in the Methods section). Similar to ACA loss in regression, the final ACA loss in classification is a combination of classification loss and contrast loss.

We compared our method on three benchmark MMP datasets for AC classification against fingerprint-based ML models (MLP, LightGBM, XGBoost, and RF) and the AC-agnostic ACGCN model. The results show that AC-informed ACGCN model significantly outperforms existing competitive fingerprint-based ML models in AC classification^20, 21^, and also significantly outperforms the AC-agnostic ACGCN^31^ model across all evaluation metrics on all three benchmark datasets. Specifically, AC-informed model improves the MCC of AC-agnostic model from 0.373 to 0.462 on the Melanocortin receptor 4 dataset, from 0.451 to 0.550 on the Mu opioid receptor dataset, and from 0.487 to 0.510 on the Thrombin dataset (Supplementary Table S9). The results show that the AC-awareness concept can be further extended to AC classification in MMP datasets, demonstrating its versatility and robustness in different predictive modeling tasks within cheminformatics.

### AC-awareness improves prediction of pharmacokinetic and safety properties.

To explore AC-awareness beyond activity prediction, we evaluated ACANET for prediction of relative changes across ten ADMET properties (Supplementary Table S4). An established approach, DeepDelta^38^, predicts ADMET property differences by directly training on molecular pairs and their property differences, outperforming both ECFP-based RF and LightGBM models. Another widely used approach, ChemProp^39^, trains a GNN model using the absolute ADMET property values. Given our method’s significant improvements in predicting absolute activity values, we trained ACANET on absolute property values and inferred the relative changes (delta values) from the predicted absolute property values. We then compared ACANET’s delta values with existing methods.

Similar to activity prediction, we found that the ACA loss hyperparameters cl,cu, and α are crucial for model performance in ADMET property prediction. This is evidenced in the Caco-2 Permeability dataset (Supplementary Figure S4), underscoring the effectiveness of AC-awareness. Using the same data split and performance measurement method, ACANET demonstrated comparable performance to ChemProp^39^ and DeepDelta^38^ on the three absorption datasets, despite the more challenging task of predicting absolute ADMET property values. Moreover, ACANET achieved the lowest cross-validation RMSE across all other seven DMET datasets—two distribution datasets, one excretion dataset, three metabolism datasets, and one toxicity dataset—as shown in Supplementary Figure S8.

These results demonstrate that directly training on and predicting absolute property values to infer property differences can match or exceed the performance of state-of-the-art methods that train and predict directly on property differences. This success is attributed to the AC-awareness in ACANET, which enables the model to learn relative molecular differences in the latent space while training on absolute values. This approach provides a principled approach compared to models trained on relative differences. Our results indicate that AC-awareness is effective across molecular property predictions and shows competitive performance in indirectly predicting property differences.

## Discussion

Activity cliffs (ACs) and molecular property cliffs (PCs) pose a considerable challenge for QSAR studies^19, 21, 21, 24, 28, 37^. We introduced the concept of AC-awareness in molecular activity prediction and implemented it through the design of the ACA loss for the GNN-based ACANET model. This ACA loss improves the model’s sensitivity to ACs by combining a standard regression loss in the label space with a triplet contrastive learning loss in the latent space. The triplet contrastive learning loss is designed to be sensitive to activity cliffs. At the core of this approach are High-Value Activity Cliff Triplets (HV-ACTs), which consist of an anchor molecule, a positive molecule with comparable activity, and a negative molecule that exhibits a significantly different activity level. HV-ACTs expose suboptimal latent space distributions, where molecules that should be clearly distinguished based on their activity levels are incorrectly positioned close together. By optimizing the relative distances of these triplets in the latent space, the ACA loss ensures that the model can effectively distinguish between molecules that are structurally similar but have vastly different activities while also bringing closer together molecules that are structurally different but have similar activities. This optimization aligns the relative distances of HV-ACTs in the latent space with the relative differences in their activity labels, resulting in a more coherent and biologically relevant representation.

Our results showed that during model training, the standard regression loss cannot optimize HV-ACTs in the latent space, as evidenced by the fact that the number of HV-ACTs does not decrease during training. However, when AC-awareness is infused into the model, it effectively addresses this limitation by gradually reducing the number of mined HV-ACTs. This enhancement allows the model to become more sensitive, enabling it to better distinguish between structurally similar molecules with vastly different activities and recognize similarities between structurally different molecules with comparable activities. The comprehensive benchmark results showed that GNN models using MAE loss with AC-awareness significantly outperform those without AC-awareness on both low-sample LSSNS and higher-sample HSSMS datasets, achieving an average performance improvement of 6.59% to 7.16%. This consistently strong performance across diverse dataset types underscores the robustness of AC-awareness in enhancing model activity predictions, regardless of dataset size or scaffold diversity. Furthermore, beyond activity prediction, our preliminary results indicate that AC-awareness also benefits the ACANET model in predicting other molecular properties, such as ADMET. Additionally, we extended the ACA loss from regression tasks to classification tasks, demonstrating its effectiveness in predicting activity cliffs in MMP datasets. This suggests that the proposed cliff-awareness is a versatile concept that can potentially be applied to other tasks in molecular deep learning.

While our results demonstrate the effectiveness of AC-awareness in enhancing GNN models for molecular activity prediction, the performance improvements observed with AC-awareness may vary depending on the specific characteristics of the dataset, such as the degree of scaffold diversity or the prevalence of activity cliffs. This suggests that while AC-awareness is robust, its applicability might require careful tuning of hyperparameters like the α factor for different datasets. Additionally, the current implementation of AC-awareness is primarily focused on regression tasks. Although we successfully extended ACA loss to classification tasks in this study, further exploration is needed to fully understand its potential across a broader range of molecular property prediction tasks, including those involving more complex or multi-task learning scenarios. Moreover, while we have demonstrated the utility of AC-awareness in both activity and ADMET property predictions, future studies could explore its impact on other critical molecular properties, such as drug-likeness, toxicity prediction, or even protein-ligand binding affinity. In principle, broadening the application of property-cliff awareness to these additional areas could significantly enhance the performance of molecular deep learning models in various stages of drug discovery, from early screening to late-stage optimization.

## Online Methods

### Datasets

#### Molecular property prediction datasets.

We consider a total of 52 datasets, including 9 LSSNS activity regression datasets, 30 HSSMS activity regression datasets, 3 MMP activity cliff classification datasets, and 10 ADMET delta regression datasets (Figure 2).

The 9 LSSNS datasets (Supplementary Table S1, Supplementary Figure S1a) contain small molecules with relatively conserved scaffold (similar structures) but varying activity labels, with sample sizes ranging from less than 50 to over 100 compounds (3 sets are less than 50 compounds, three sets are in 50–100, 3 sets are greater than 100 compounds). The LSSNS datasets were collected from fragment-to-lead medicinal chemistry publications in 2019^34^ and the ChEMBL^41^ database. These datasets represent a real-world scenario of a series of conservative compounds developed by a single laboratory or institution (Supplementary Table S1), and we use them to test the ACA loss in the low-sample size and narrow scaffold regimes.

The 30 HSSMS benchmark datasets (Supplementary Table S2) were curated by van Tilborg *et al*. (2022)^19^ using data from ChEMBL^41^. It serves as a benchmark for exposing the limitations of molecular machine learning with activity cliffs (ACs). The HSSMS consists of 30 mixed-scaffold datasets totaling 48,707 molecules. The HSSMS datasets cover a wider range of sample sizes (from 615 to 3,657 compounds), scaffold diversity, bioassay types, and target families. Each dataset of HSSMS has been split into train and test sets, and the AC compounds are labeled with a cliff flag for evaluation purposes^19^.

The 3 MMP benchmark datasets (Supplementary Table S3) were curated by Park *et al*. (2022)^31^ for AC classification, focusing on the targets thrombin, Mu opioid receptor, and melanocortin receptor 4. The binary AC labels of MMPs are defined as MMP-cliff (AC) if $\Delta pK_{i}\geq 2$, and MMP-nonCliff (non-AC) if $\Delta pK_{i}\leq 1$.

The 10 ADMET benchmark datasets (Supplementary Table S4) were collected and preprocessed by Fralish *et al*. (2023)^38^ for delta prediction of molecular ADMET properties. These datasets include 3 for absorption properties (solubility, Caco2 permeability, and free solvation), 2 for distribution properties (fraction unbound in brain, volume of distribution at steady state), 1 for an excretion property (renal clearance), 3 for metabolism properties (microsomal clearance, hepatic clearance, and half-life), and 1 for hemolytic toxicity property. The structures were sanitized, and the property labels were log-transformed except for the free solvation dataset^38^.

#### Molecular activity labels.

In this study, the molecular activity is represented by the standard pChEMBL value (y), which is defined as follows: $-{\text{log}}_{10}$ (molar IC50, XC50, EC50, AC50, Ki, Kd or Potency)^42^, i.e., can be represented as the pIC50 values if the activity type is IC50. Given two compounds i and j, the cliff between the two compounds in the same activity type can be represented as L1 distance of the standard pChEMBL value y:

(1) $$\begin{array}{l} y_{i}=-{\text{log}}_{10}\left(x_{i}\times 10^{-9}\right),\\ y_{j}=-{\text{log}}_{10}\left(x_{j}\times 10^{-9}\right),\\ \text{cliff}=\left|y_{i}-y_{j}\right|=\left|{\text{log}}_{10}\left(\frac{x_{i}}{x_{j}}\right)\right|, \end{array}$$

where $x_{i}$ and $x_{j}$ are the activity values with nanomolar unit, $y_{i}$ and $y_{j}$ are the standard pChEMBL values. As a result, the fold change in nanomolar unit FC(nmol) is ten to the cliff power:

(2) $$FC(\text{nmol})=\frac{x_{j}}{x_{i}}=10^{\text{cliff}}$$

Therefore, the activity fold change (FC) between two compounds for cliff values 0.3, 1, and 2 is roughly 2, 10, and 100 times the change in nanomolar units, respectively.

#### Molecular activity cliffs in the chemical and model latent spaces.

Molecular activity cliffs (ACs) are formed by pairs of structurally similar compounds that are active against the same bio-target but have large differences in potency^19,29^. Importantly, two aspects complicate the consistent assessment of ACs in computational and medicinal chemistry: the definition of “similarity” and the quantification of “large” potency differences^29^.

Conventionally, Extended Connectivity Fingerprints (ECFPs) are used to measure structural Tanimoto similarity between compounds in chemical space^43,44^. However, similarity can also be assessed in latent space through molecular representations learned by GNN models using graph convolutions. Ideally, unlike the original chemical space, the presence of ACs should be significantly reduced in the latent space of molecular representations. This is because the molecular representation in the latent space should align with the activity labels of the molecules, as illustrated in Figure 3a, allowing for better predictions. Therefore, optimizing the similarity between compounds in the latent space is a crucial step in addressing the molecular activity cliff challenges in deep learning.

Furthermore, the magnitude of the bioactivity difference that defines an activity cliff (AC) is subjective and can vary depending on the target and the assay used for measuring activity. Different studies may have different thresholds, such as a 10-fold^19^ or 100-fold^29^ difference (i.e., reported Ki, IC50, or EC50 values) in bioactivity to define AC pairs. However, determining ACs requires careful consideration of dataset characteristics, such as the chemical series being studied and the distribution of bioactivity values. In this study, instead of fixing a specific threshold for defining ACs, we introduced the cliff hyperparameters “cliff lower” (cl) and “cliff upper” (cu) that can be tuned to control the number of identified activity cliff triplets (ACTs). This approach allows for more flexibility in defining ACs and can be tailored to the specific dataset being analyzed. More details about the ACTs mining by cliff parameters can be found in the section on online mining of the ACTs.

#### Online mining of the activity cliff triplets (ACTs).

In this study, we employed online mining of activity cliff triplets (ACTs) for model contrastive learning in the latent space. Online ACT mining offers computational efficiency and scalability compared to offline mining, as it dynamically selects ACTs in each training batch based on the model’s current state^45^. During the mining process, we identify a positive compound and a negative compound for each anchor compound to form an ACT. The positive compound has an activity value close to the anchor, while the negative compound exhibits significantly different activity. To facilitate real-time conditional ACT sampling in each batch, we introduce “cliff lower” (cl) and “cliff upper” (cu) as cliff cut-off hyperparameters (Figure 1b). By comparing the activity difference between compounds and the anchor, we categorize compounds as positive examples if the difference is within the “cliff lower” threshold (cl) and as negative examples if the difference exceeds the “cliff upper” threshold (cu). When cl is equal to cu, the triplets are obtained using a single “cliff” hyperparameter, maximizing the number of ACTs acquired. It is important to note that cl should be smaller than cu, and leaving a suitable margin ensures the obtained triplets are reasonable.

To obtain highly valuable ACTs (HV-ACTs) in each batch, only an ACT with its triplet loss greater than zero is counted (Figure 1b). This ensures that the selected triplets are informative and contribute effectively to the model’s learning. The number of mined HV-ACTs reflects the degree of ACs in the current stage of molecular representations. If the model is already AC-aware during training, the number of mined HV triplets should gradually decrease as training progresses. This reduction occurs because the AC-awareness of the model optimizes the latent feature space to reduce prediction errors. Gradually decreasing the number of mined HV-ACTs helps the model focus on refining the latent feature space for compounds that are more challenging to predict accurately.

### Activity cliff awareness (ACA) loss function

#### 1) The overall definition of ACA loss in regression task.

The activity cliff awareness (ACA) loss ${ℒ}_{aca}$ is a combination of the regression loss (e.g., mean absolute error MAE loss) and the triplet contrastive loss (Figure 1a). Specifically, in each batch, the MAE loss ${ℒ}_{\text{mae}}$ was computed as the mean absolute difference between the predicted and actual activity values for the compounds in the batch. The triplet contrastive loss ${ℒ}_{tsm}$ was then computed based on HV-ACTs of compounds within the batch, where a triplet consists of an anchor compound, a positive compound with similar activity (controlled by parameter cl), and a negative compound with dissimilar activity (controlled by parameter cu). The triplet contrastive loss encourages the model to learn representations that distinguish between the positive and negative compounds while also maintaining proximity between the query and positive compounds. The ACA loss was then defined as the sum of the regression MAE loss and a cliff awareness factor α multiplied by the triplet contrastive loss:

(3) $${ℒ}_{aca}={ℒ}_{mae}+\alpha *{ℒ}_{tsm},$$

(4) $${ℒ}_{mae}=\frac{1}{N}\sum_{i=1}^{N}\left|y_{i}-{\overset{ˆ}{y}}_{i}\right|,$$

(5) $$\begin{array}{c} {ℒ}_{tsm}=\frac{1}{M^{\prime }}\sum_{j=1}^{M^{\prime }}{\left[d\left(A_{j},P_{j}\right)-d\left(A_{j},N_{j}\right)+m_{j}\right]}_{+}=\frac{1}{M^{\prime }}\sum_{j=1}^{M^{\prime }}{\left[{\left\|f_{j}^{A}-f_{j}^{P}\right\|}_{2}-{\left\|f_{j}^{A}-f_{j}^{N}\right\|}_{2}+m_{j}\right]}_{+}, \end{array}$$

where the ${ℒ}_{\text{mae}}$ is the MAE loss, the ${ℒ}_{tsm}$ is the triplet loss with soft margin, the awareness factor α is tunable parameter that determines the relative importance of the triplet contrastive loss compared to the MAE loss. N is batch size, while $M^{\prime }$ is the number of the mined high valuable ACT (HV-ACT) in each batch. The $y_{i},\overset{ˆ}{y_{i}}$, and $f_{i}$ are the i-th true label, predicted label and latent vectors, respectively. In the triplet loss with soft margin (TSM) ${ℒ}_{tsm}$, the distances between Anchor-Positive (A-P) or Anchor-Negative (A-N) pairs are calculated using the latent space vectors f and the L2-norm distance. The item $m_{j}$ represents the soft margin of the mined j-th triplet.

#### 2) The soft margin of the triplet contrastive loss.

The soft margin $m_{j}$ was calculated as the difference between the ground-truth L2-norm distances of A-N and A-P pairs, i.e.,

(6) $$m_{j}=d_{GT}\left(A_{j},N_{j}\right)-d_{GT}\left(A_{j},P_{j}\right)=\left|y_{j}^{A}-y_{j}^{N}\right|-\left|y_{j}^{A}-y_{j}^{P}\right|,j\in M^{\prime }.$$

As the prediction task is one-dimensional, meaning that each sample has only one associated activity label, the ground-truth distance between A-N and A-P pairs can be computed using either the L1-norm or L2-norm. In this scenario, since the distance is determined by the ground-truth activity labels, both L1-norm and L2-norm will yield identical results. By using the soft margin, we can see that when $d\left(A_{j},P_{j}\right)$ equals to $d_{GT}\left(A_{j},P_{j}\right)$ and $d\left(A_{j},N_{j}\right)$ equals to $d_{GT}\left(A_{j},N_{j}\right),{ℒ}_{\text{aca}}$ equals to zero, which means that when the relative distances of (A, P, N) in the latent space equals to the relative distances of (A, P, N) in the ground truth space, the loss is zero and no gradients generated. The soft margin, $m_{j}$, was introduced to ensure that the loss function is robust to the relative distances of different triplets (Figure 1c). The soft margin in triplet loss is a more flexible and robust training method that allows for a degree of tolerance in the similarity or dissimilarity of triplets.

#### 3) The squared ACA loss.

The squared ACA loss is a variation of the ACA loss that incorporates the mean squared error (MSE) loss as the regression loss and a squared triplet loss term. This modification helps the model adhere to the AC principle. The formula for the squared ACA loss is:

(7) $${ℒ}_{aca}^{2}={ℒ}_{mse}+\alpha *{ℒ}_{tsm}^{2},$$

where ${ℒ}_{mse}$ is the mean squared error loss, defined as: Here, $y_{i}$ is the true label of the i-th sample, and ${\overset{ˆ}{y}}_{i}$ is the predicted label for the i-th sample. The second term, ${ℒ}_{tsm}^{2}$, is the squared triplet loss, defined as:

(8) $${ℒ}_{tsm}^{2}=\frac{1}{M^{\prime }}\sum_{j=1}^{M^{\prime }}{\left[{\left\|f_{j}^{A}-f_{j}^{P}\right\|}_{2}^{2}-{\left\|f_{j}^{A}-f_{j}^{N}\right\|}_{2}^{2}+m_{j}^{2}\right]}_{+}.$$

Here, $f_{j}^{A},f_{j}^{P}$, and $f_{j}^{N}$ are the feature embeddings of the anchor, positive, and negative in the j-th triplet. The soft margin term $m_{j}^{2}$ is defined as:

(9) $$m_{j}^{2}={\left|y_{j}^{A}-y_{j}^{N}\right|}^{2}-{\left|y_{j}^{A}-y_{j}^{P}\right|}^{2},j\in M^{\prime }.$$

The squared triplet loss is designed to have the same scale as the MSE loss, allowing them to be combined in a meaningful way. By adjusting the “squared” parameter in the ACA loss, one can choose between using the standard ACA loss or its squared version.

#### 4) The tuneable hyperparameters in ACA loss.

The ACA loss has several tuneable hyperparameters that can be adjusted to improve its performance based on the specific task and dataset.

- α: This is a scalar value that controls the relative weight of the regression loss and the contrastive loss. Higher values of α give more weight to the triplet loss, while lower values give more weight to the regression loss. The default value is 0.1.
- cliff lower (cl): This hyperparameter sets the lower threshold for the AC, which is the difference between the activity of the anchor compound and the activity of the positive compound in an ACT. If the difference is less than or equal to cl, the compounds will be considered as the positive compound in the ACT. The default value is 1.0.
- cliff upper (сu): This hyperparameter sets the upper threshold for the AC, which is the difference between the activity of the anchor compound and the activity of the negative compound in an ACT. If the difference is greater than cu, the compounds will be considered as the negative compound in the ACT. The default value is 1.0.
- squared: This is a boolean value that determines whether the squared version of the triplet loss (combined with MSE loss) should be used or not. The default value is False.
- p-norm: This hyperparameter sets the norm used for the distance metric between the anchor, positive, and negative embeddings in the triplet loss. By default, the ACA loss uses the Euclidean norm (p = 2), but other values of p can also be used.

The optimal values of these hyperparameters may vary depending on the specific task and dataset. As illustrated in Supplementary Figure S1b, the cliff parameters corresponding to the maximum number of mined conditional triplets vary across different datasets, reflecting differences in the distribution of activity labels. Therefore, it is advisable to perform hyperparameter tuning to determine the best values for each specific task and dataset.

### ACANET model architecture and training

We investigated four widely-used GNN backbones in the ACANET: Graph Convolutional Network (GCN)^46^, Graph Isomorphism Network (GIN)^47^, Graph Attention Network (GAT)^48^, and Principal Neighborhood Aggregation (PNA)^49^. PNA was selected as the default backbone due to its reported superior performance in related tasks^49^. The message passing settings for these convolutional layers were tailored to each backbone’s architecture. For the GCN backbone, a mean aggregation approach was used to combine information from neighboring nodes. In the GAT backbone, a multi-head attention mechanism was employed, where outputs from multiple attention heads were averaged to form the final node representation. For the GIN layers, a trainable parameter ϵ was used to adjust the weight of the central node’s feature during aggregation, allowing for flexibility in balancing the influence of neighboring nodes. In the PNA backbone, multiple aggregators (‘mean’, ‘min’, ‘max’, ‘sum’, ‘std’) and scalers (‘identity’, ‘amplification’, ‘attenuation’) were employed to extract node features from various neighborhoods. All these GNN convolutional layers were implemented using the PyTorch Geometric^50^ library, ensuring efficient computation and integration within the ACANET model.

The inputs to ACANET are small molecules with graph representations (Figure 1a), which are featurized using the 39 node features and 10 edge features that described in Supplementary Table S5. The graph convolutional layers are with channel size of (64, 128, 256, 512). The dense layers followed a pyramid-shaped structure with 256, 128, and 32 neurons, respectively, allowing the network to learn complex representations in the early layers and progressively reduce the dimensionality towards the output layer. The default ACANET configuration did not include dropout, while Batch Normalization was applied after each graph convolutional layer to stabilize the learning process. A Global Max Pooling (GMP) layer was employed to retain the most important features from the graph, selecting the maximum value in each feature map channel(Figure 1a).

The outputs of ACANET included the predicted activity labels and the latent vectors derived from the GMP layer, which were used for the distance calculation of activity cliff triplets (ACTs) as they encapsulate the most relevant information from the molecular graph. For training ACANET, a learning rate of 1e-4 was fixed, and the batch size was set to 128 for all datasets. The batch size is particularly important in ACANET, as a larger batch size enables more effective sampling of high-value activity cliff triplets (HV-ACTs). An early stopping mechanism was implemented to select the best model based on the ACA loss value calculated from the monitor dataset, ensuring optimal model performance.

### ACANET with and without AC-awareness across different GNNs

To verify the concept of AC-awareness and test the effect of ACA loss, we analyzed ACANET with and without AC-awareness across four different backbones (GCN, GIN, GAT, and PNA). A medium-sized dataset PPARδ (n = 1125) from the HSSMS collection was used, and it was split into training, validation, and test sets in a proportion of 6:2:2. The ACANET models with AC-awareness (MAE loss + TSM loss) and without AC-awareness (MAE loss only) used the exact same hyperparameters except for the loss function. For the hyperparameters in the ACA loss, the awareness factor α and cliff parameters (cu and cl) were all set to 1, following the default settings. We recorded the training history of MAE loss, the number of mined HV-ACTs ${(M}^{\prime })$ during training, and the RMSE performance of the validation and test sets over all training epochs. To improve the robustness of the results, we repeated the data splits 10 times with different random seeds, resulting in 10 repetitions of the PoC experiments on these different splits. The final results for PNA-based ACANET are shown in Figure 3c, with shaded areas representing the standard deviation from 10 repetitions with different random seeds in the data splits. The impact of AC-awareness across the four different GNN backbones is illustrated in Figure 4, which shows the number of mined HV-ACTs $\left(M^{\prime }\right)$ for the training, validation, and test sets during training, as well as the final test RMSE performance. The best model was selected based on the validation set RMSE (Supplementary Table S6).

### Ablation studies on the ACA loss

Ablation studies were conducted to investigate the impact of the AC-awareness factor α and cliff hyperparameters in the ACA loss on PNA-based ACANET performance using the BRAF dataset from LSSNS with a stratified five-fold cross-validation repeated at least three times (Figure 5).

In the first study, the cliff lower (cl) and cliff upper (cu) parameters were varied while keeping α fixed at 1. The range of values for cl and cu was 0 to 4 with a step size of 0.1, ensuring that cl was less than or equal to cu. This study evaluated the effect of these changes on the number of mined conditional ACTs (M) and the RMSE performance of ACANET. The validation RMSE was averaged from five-fold cross-validation. The cliff parameters control the number of mined conditional ACTs (M), which are crucial for predicting molecular activity. The evaluation on other four LSSNS datasets (PLK1, IDO1, USP7, and RIP2) are shown in Supplementary Figure S2.

The second study focused on the AC-awareness factor α in the ACA loss. Here, the cl and cu parameters were fixed, and α was varied from 0.00 (no AC-awareness) to 0.01, 0.05, 0.10, 0.20, 0.50, and 1.00. This study evaluated the historical performance of ACANET over at least 800 training epochs under different α values using the BRAF dataset from LSSNS with five-fold cross-validation repeated at least three times. The results provide insights into the impact of the AC-awareness factor α on ACANET performance and can guide the selection of appropriate values for this parameter in future applications.

### Analysis of label incoherence across chemical and latent spaces

We conducted both qualitative and quantitative analyses on a narrow scaffold BRAF dataset to examine the discontinuity (or cliff index) of compound activity distribution in the original chemical space and the latent space learned by our model.

#### Qualitative analysis.

In the original chemical space, we computed ECFP4 fingerprints (1024-bit) to measure the Tanimoto distance between molecules and used TMAP^51^ to visualize the chemical space in 2D (Figure 6a). For the latent space, we performed PCA to obtain 2D embeddings using the learned 512-dimensional feature vectors from the model, both with and without ACA-awareness (Figure 6b & c). In these 2D spaces, molecules were colored by their pIC50 activity labels to visualize the local connectivity of activities in different spaces.

#### Quantitative analysis.

To quantitatively assess label incoherence (or discontinuity) in different spaces, we introduced a new metric called the Label Incoherence Index (LII), which measures the average local discrepancy of molecular activity labels in a given space. The LII was calculated for molecular representations using the following steps:

##### 1) Finding k-nearest neighbors using cosine distance:

For each molecular representation $x_{i}$ in X, find the k-nearest neighbors using cosine distance:

$$d_{\text{cosine}}\left(x_{i},x_{j}\right)=1-\frac{x_{i}\cdot x_{j}}{\left\|x_{i}\right\|\left\|x_{j}\right\|}$$

Let ${𝒩}_{i}$ denote the set of indices of the k-nearest neighbors for the data point $x_{i}$, excluding $x_{i}$ itself.

##### 2) Overall label incoherence index calculation:

Compute the mean of the average absolute differences in labels y across all data points to obtain the Label Incoherence Index (LII):

$$LII=\frac{1}{n}\sum_{i=1}^{n}\left(\frac{1}{k}\sum_{j\in {𝒩}_{i}}\left|y_{j}-y_{i}\right|\right)$$

where n is the total number of data points, $y_{i}$ is the label of the i-th data point, and $y_{j}$ is the label of its j-th nearest neighbor.

We calculated the LII for the 512-dimensional latent space vectors of models with ACA-awareness and models without ACA-awareness. This comparison allowed us to evaluate the coherence of features and labels in the latent spaces of both types of models. Additionally, we computed the LII for the ECFP fingerprint representations of the structures to evaluate label coherence in the original structural space, the LII for the original fingerprint representations provided a baseline for comparison.

### Analysis of AC-awareness on LSSNS datasets

To evaluate the effectiveness of ACA-awareness on molecular activity prediction, we conducted a benchmark study on the LSSNS collection using both MAE and MSE as the regression loss (Supplementary Table S7). We trained two versions of the same model: one with ACA loss and the other without. For the model with ACA loss, we tuned the parameters of cliff lower (cl), cliff upper (cu), and ACA factor a. Initially, we fixed the awareness factor a at its default value of 0.1 and used a “T-shape” grid search strategy to find the optimal values of cl and cu. Specifically, we kept the cliff lower fixed at 0.1 and varied the cliff upper from 0.5 to 4.0 with a step size of 0.5. Then, we used the best cliff upper value and varied the cliff lower from 0.1 to the best cliff upper value in 8 steps. After determining the optimal cliff lower and cliff upper parameters, we tuned a by performing stratified five-fold cross-validation on the training set with a range of [0., 0.01, 0.05, 0.1, 0.2, 0.5, 1.]. The best a value was chosen based on cross-validation performance. In contrast, for the model without ACA loss, we set a to 0.0. All models were trained for 800 epochs using the same batch size (128) and learning rate (1e-4), and the best parameters of cl,cu, and a were used in the model with ACA. We selected the best RMSE value on the validation set of a stratified five-fold cross-validation and repeated the process ten times to ensure the stability of the results. We reported the average RMSE and the standard deviation performance for each model. To assess the significance of the performance improvement of the model with ACA loss, we used paired t-tests. The levels of statistical significance are indicated as follows: *p<0.05,**p<0.01,***p<0.001, and ****p<0.0001. Results not meeting these thresholds are denoted as “n.s.” for not significant.

### Benchmarking ACANET on HSSMS datasets

We assess the performance of ACANET on the HSSMS benchmark datasets. These datasets serve as a representative set of mixed-scaffold datasets for exposing the limitations of molecular machine learning with ACs^19^. To compare the benchmark results with those obtained using off-the-shelf machine learning models for molecular activity prediction, we compared AC-awareness equipped ACANET with the following models: the graph-based GAT, GCN, AFP, and MPNN methods (without AC-awareness); the SMILES-based Transformer, CNN, and LSTM; and ECFP4 fingerprint-based MLP, KNN, GBM, RF, and SVM. The statistical significance of the performance difference between ACANET with AC-awareness and the off-the-shelf models was determined using the paired t-test with a significance level of 0.05. Additionally, we performed a paired t-test to assess the statistical significance of the performance improvement of ACANET with AC-awareness compared to ACANET without AC-awareness.

To train ACANET on all datasets, we used a batch size of 128 and a learning rate of 1e-4. For hyperparameter optimization in ACA loss, we followed these steps: First, we fixed the awareness factor a at its default value of 0.1 and used a “T-shape” grid search strategy to find the optimal values of cliff lower and cliff upper (Supplementary Figure S3a). Specifically, we kept the cliff lower fixed at 0.1 and varied the cliff upper from 0.5 to 4.0 with a step size of 0.5. Then, we used the best cliff upper value and varied the cliff lower from 0.1 to the best cliff upper value in 8 steps. After determining the optimal cliff lower and cliff upper parameters, we tuned a by performing stratified five-fold cross-validation on the training set with a range of [0., 0.01, 0.05, 0.1, 0.2, 0.5, 1.] (Supplementary Figure S3b). The best a value was chosen based on training set cross-validation performance (Supplementary Figure S5), the training set cross-validation performance of ACANET model with and without AC-awareness is shown in Supplementary Table S8. We set all other parameters in ACA loss, such as squared (squared = False) and p-norm (p = 2), to their default values. During the final training stage of ACANET, we employed early stopping based on the validation set in a nested stratified five-fold cross-validation to select the best model. To predict on the external test set, we averaged the predictions from the five sub-models from the nested cross-validation. The performance was evaluated using the RMSE metric on the overall test set $\left(RMSE_{\text{all}}\right)$ and cliff-specific test set $\left(RMSE_{\text{cliff}}\right)$ as used by van Tilborg *et al*.^19^. The results are shown in Supplementary Figure S6.

### Benchmarking ACANET on ADMET datasets

To further evaluate our method for predicting other molecular properties such as ADMET, we tested ACANET on the 10 ADMET benchmark datasets (Supplementary Table S4) for property delta prediction. As molecular property delta (the property difference between two molecules) prediction can assess model performance on “property cliffs”, we compared the model performance with established methods such as ECFP-based RF and LightGBM ML models, and the GNN-based ChemProp and DeepDelta models for ADMET property differences prediction. To predict the delta values using our method, we trained the ACANET model on the absolute ADMET properties and inferred the property differences on the test set by predicting the absolute values. During training, the ACA loss hyperparameters cl,cu, and α were optimized using the same strategy as described in the HSSMS benchmarking. An example of hyperparameter search for the Caco-2 Permeability dataset is shown in Supplementary Figure S4. Using the same data split method and delta prediction evaluation as the established methods, the final model performance of delta RMSE from the 5 × 10-fold cross-validation results is shown in Supplementary Figure S8.

### Extending AC-awareness for activity cliff classification

In addition to QSAR, predicting pairs of AC molecules is another important challenge related to activity cliffs (AC). This task involves classifying whether a pair of similar compounds forms an AC or not^6, 30, 31^. In this section, we extend AC-awareness to AC classification in matched molecular pairs.

An AC pair can be represented by a matched molecular pair (MMP), where the two molecules share the same core structure but have different substituents. Following Park *et al*.^31^ and Tamura *et al*.^37^, molecule pairs were decomposed using the algorithm by Hussain *et al*.^52^, with each fragment treated as a subgraph. The representation of the molecule is the concatenation of its core and substituent representations encoded by GNN. Most deep learning methods currently use cross-entropy loss for AC prediction, without explicitly emphasizing the substituents that contain rich SAR knowledge. To develop AC-awareness for classification tasks, these AC pairs have already been pre-provided and do not need to be constructed in the batch. We use a simple yet effective contrastive learning strategy to push the features of ACs apart and bring the features of non-ACs closer. Specifically, for a given MMP consisting of molecule A and molecule B, we calculate the paired contrastive loss $\left({ℒ}_{pcl}\right)$ to maximize the distance between A and B if the MMP is an AC, and minimize the distance otherwise:

(10) $${ℒ}_{pcl}=\sum_{i=1}^{M}y_{mmp}\cdot \left(1+\text{sim}\left(f_{i}^{A},f_{i}^{B}\right)\right)+\left(1-y_{mmp}\right)\cdot \left(1-\text{sim}\left(f_{i}^{A},f_{i}^{B}\right)\right),$$

where $y_{mmp}$ is the label of the AC pair, and $f_{i}^{A}$ and $f_{i}^{B}$ are the representations of molecules A and B, respectively. The final loss is given by:

(11) $$ℒ={ℒ}_{cls}+\alpha \cdot {ℒ}_{pcl},$$

where α is the AC-awareness factor balancing the classification loss and contrastive loss. We conducted experiments on three different MMP benchmark datasets: thrombin, mu opioid receptor, and melanocortin receptor4. The best factor α was optimized (thrombin: 0.1, Mu Opioid Receptor: 0.4, melanocortin receptor 4: 0.5) based on the cross-validation performance of the training set. Using the same data splits and evaluation metrics, we compared the performance of GNN-based ACGCN^31^ models (with and without AC-awareness) against fingerprint-based ML models (MLP, LightGBM, XGBoost, and RF), as summarized in Supplementary Table S9.

## Data Availability

All data used in the paper, including the 9 small-sample size narrow scaffold (LSSNS) datasets, the 30 large-sample size mixed scaffold (HSSMS) datasets, the 3 MMP datasets and 10 ADEMT datasets are released at https://github.com/bidd-group/MPCD. In addition, datasets are being made available via the new Molecular Property Cliff Task (https://tdcommons.ai/single_pred_tasks/MPC/) in Therapeutics Data Commons^40^ (https://tdcommons.ai).
